# Evidence for European presence in the Americas in ad 1021

**DOI:** 10.1038/s41586-021-03972-8

**Published:** 2021-10-20

**Authors:** Margot Kuitems, Birgitta L. Wallace, Charles Lindsay, Andrea Scifo, Petra Doeve, Kevin Jenkins, Susanne Lindauer, Pınar Erdil, Paul M. Ledger, Véronique Forbes, Caroline Vermeeren, Ronny Friedrich, Michael W. Dee

**Affiliations:** 1 Centre for Isotope Research, University of Groningen, Groningen, the Netherlands; 2 Parks Canada Agency, Government of Canada, Dartmouth, Nova Scotia Canada; 3 Laboratory for Dendrochronology at BAAC, ’s-Hertogenbosch, the Netherlands; 4 Cultural Heritage Agency of The Netherlands, Amersfoort, the Netherlands; 5 Curt-Engelhorn-Center Archaeometry, Mannheim, Germany; 6 Department of Archaeology, Queens College, Memorial University of Newfoundland, St Johns, Newfoundland Canada; 7 Department of Geography, Memorial University of Newfoundland, St Johns, Newfoundland Canada; 8 BIAX Consult, Zaandam, the Netherlands

**Keywords:** Archaeology, Archaeology, Plant physiology, Mass spectrometry

## Abstract

Transatlantic exploration took place centuries before the crossing of Columbus. Physical evidence for early European presence in the Americas can be found in Newfoundland, Canada^1,2^. However, it has thus far not been possible to determine when this activity took place^3–5^. Here we provide evidence that the Vikings were present in Newfoundland in ad 1021. We overcome the imprecision of previous age estimates by making use of the cosmic-ray-induced upsurge in atmospheric radiocarbon concentrations in ad 993 (ref. ^6^). Our new date lays down a marker for European cognisance of the Americas, and represents the first known point at which humans encircled the globe. It also provides a definitive tie point for future research into the initial consequences of transatlantic activity, such as the transference of knowledge, and the potential exchange of genetic information, biota and pathologies^7,8^.

## Abstract (web summary)

Precise dating of wooden artefacts at a Norse settlement in Newfoundland establishes that the Norse were in the Americas in ad 1021.

## Main

**Extended Data Fig. 1:**
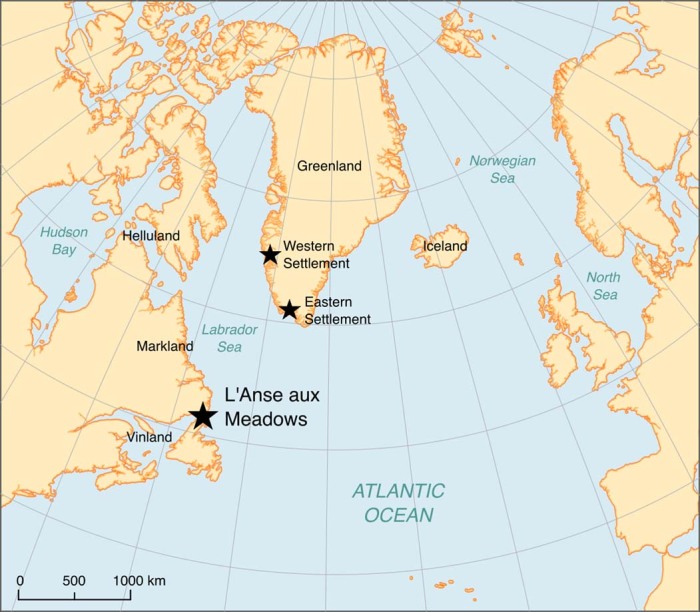
North Atlantic regions explored by the Norse. LAM lies on the Northern Peninsula of Newfoundland. The map shows the main settlements on Greenland from where the Norse embarked, and the regions they named Helluland, Markland and Vinland. Map: R. Klaarenbeek.

**Extended Data Fig. 2:**
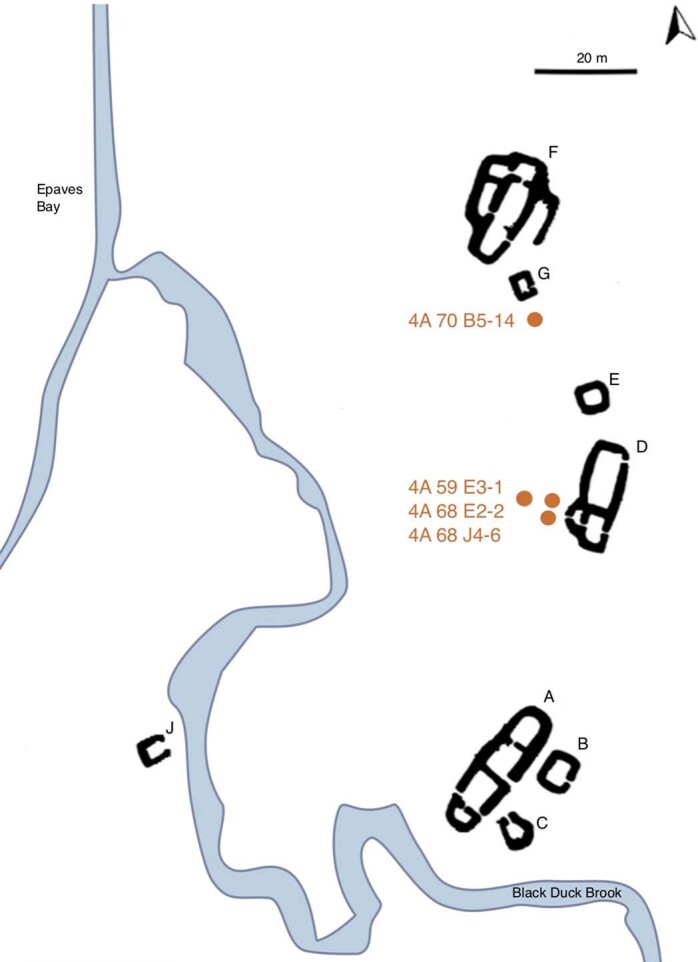
Schematic overview of the site (after Wallace 2003)^2^ and origin of our samples. Indicated are the contours of different Norse structures (A–J) and the locations (brown) at which the wood items were found that are used in the current study.

The Vikings (or Norse) were the first Europeans to cross the Atlantic^9^. However, the only confirmed Norse site in the Americas is L’Anse aux Meadows, Newfoundland^9–12^ (Extended Data Figs. 1 and 2). Extensive field campaigns have been conducted at this UNESCO (United Nations Educational, Scientific, and Cultural Organization) World Heritage Site, and much knowledge has been gained about the settlement and its contemporary environment^2,13–15^ (Supplementary Note 1). Evidence has also revealed that L’Anse aux Meadows was a base camp from which other locations, including regions further south, were explored^15^.

**Fig. 1:**
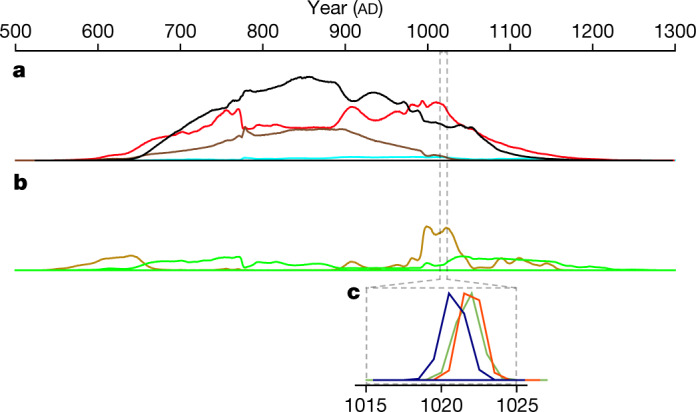
Date ranges obtained from our wiggle matches in comparison with legacy ^14^C data. **a**, **b**, Averaged probability density functions for different sample types (Extended Data Fig. 3, Supplementary Note 5 and Supplementary Data 1). **a**, Samples susceptible to inbuilt age. Light blue, whale bone (*n* = 1, uncorrected for marine reservoir effect); red, wood (*n* = 17); brown, burnt wood (*n* = 7); black, charcoal (*n* = 22). **b**, Short-lived samples. Light green, turf or sod from the Norse buildings (*n* = 4); olive, outermost rings and twigs from Norse-modified wood (*n* = 4). **c**, Wiggle-matched probability density functions for the last growth ring of each wood item. Dark green, 4A 59 E3-1; navy, 4A 68 J4-6; orange, 4A 68 E2-2. Source data

**Extended Data Fig. 3:**
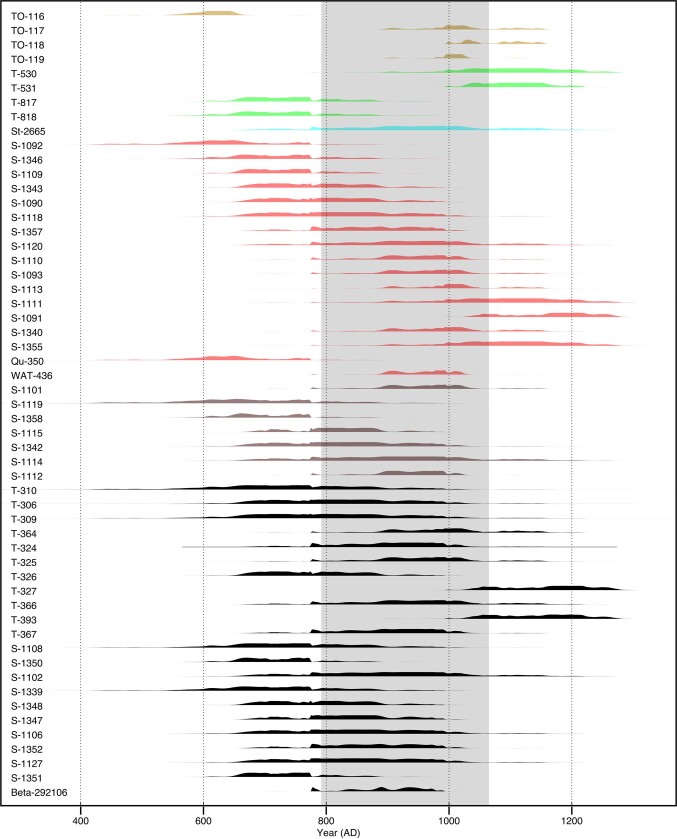
The 55 legacy ^14^C dates on Norse contexts at LAM. Samples susceptible to inbuilt age: light blue, whale bone (*n* = 1, uncorrected for Marine Reservoir Effect); red, wood (*n* = 17); brown, burnt wood (*n* = 7); black, charcoal (*n* = 22). Short-lived samples: light green, turf or sod samples from the walls of the Norse buildings (*n* = 4); olive, outermost rings and twigs from Norse-modified wood (*n* = 4). See Supplementary Data 1.

**Extended Data Fig. 4:**
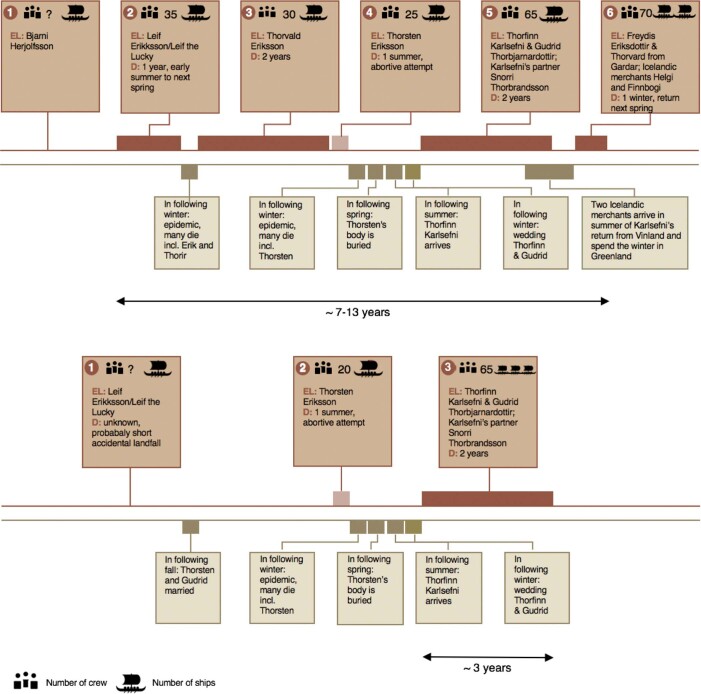
Overview of the number and order of the different voyages by the Norse to the Americas based on the information from the Sagas. Indicated for each voyage are the expedition leader (EL), the duration (D), the number of attending crew and the number of ships. Top, summary of the information from the *Saga of the Greenlanders*, which indicates that the number of winters spent at Vinland is seven. Given the short sailing seasons and the impossibility of making round trips between Greenland and Vinland in one year, the time between the first arrival of the Norse at Vinland and their ultimate return is estimated to be about thirteen years; bottom, summary of the information from the *Saga of Erik the Red*, with the estimated minimum time between the first arrival of the Norse at Vinland and their ultimate return, amounting to about three years.

The received paradigm is that the Norse settlement dates to the close of the first millennium^9^; however, the precise age of the site has never been scientifically established. Most previous estimates have been based on stylistic analysis of the architectural remains and a handful of artefacts, as well as interpretations of the Icelandic sagas, oral histories that were only written down centuries later^2,16^ (Supplementary Note 2). Radiocarbon (^14^C) analysis has been attempted at the site, but has not proved especially informative^3,17,18^. More than 150 ^14^C dates have been obtained, of which 55 relate to the Norse occupation^19^. However, the calibrated age ranges provided by these samples extend across and beyond the entire Viking Age (ad 793–1066) (Fig. 1 and Extended Data Fig. 3). This is in contrast with the archaeological evidence and interpretations of the sagas. The latter offer differing scenarios for the frequency and duration of Norse activity in the Americas, but both the archaeological and written records are consistent with a very brief occupation (Supplementary Note 3 and Extended Data Fig. 4). The unfavourable spread in the ^14^C dates is down to the limitations of this chronometric technique in the 1960s and 1970s when most of these dates were obtained. Such impediments included far greater measurement uncertainty and restrictive sample size requirements. Furthermore, many of these samples were subject to an unknown amount of inbuilt age. The term inbuilt age refers to the difference in time between the contextual age of the sample and the time at which the organism died (returned by ^14^C analysis), which can potentially reach hundreds of years. This offset was also sometimes inappropriately incorporated into summary estimates^3^.

## Cosmic radiation events as absolute time markers

In our study, we use an advanced chronometric approach to anchor Norse activity in the Americas to a precise point in time. Exact-year ^14^C results can be achieved by high-precision accelerator mass spectrometry (AMS) in combination with distinct features in the atmospheric ^14^C record^20–22^. Measurements on known-age (dendrochronological) tree rings show that ^14^C production usually fluctuates by less than 2‰ per year^23^. However, such time series have also revealed that production of the isotope rapidly increased in the years ad; 775 and ad 993 by about 12‰ (which manifests as a decrease of about 100 ^14^C yr)^24^ and about 9‰ (about 70 ^14^C yr)^6^, respectively. These sudden increases were caused by cosmic radiation events, and appear synchronously in dendrochronological records all around the world^25–29^. By uncovering these features in tree-ring samples of unknown age, it is possible to effect precise pattern matching between such samples and reference series. In so doing, if the bark edge (or more specifically, the waney edge) is also present, it becomes possible to determine the exact felling year of the tree^20^. Moreover, it is not necessary to have ^14^C dates for the outermost growth rings, because once the ring that contains the ad 993 anomaly has been detected, it simply becomes a matter of counting the number of rings to the waney edge. On the basis of the state of development of the earlywood and latewood cells in the waney edge, one can even determine the precise felling season.

## Precise dating of Norse activity in the Americas

**Extended Data Fig. 5:**
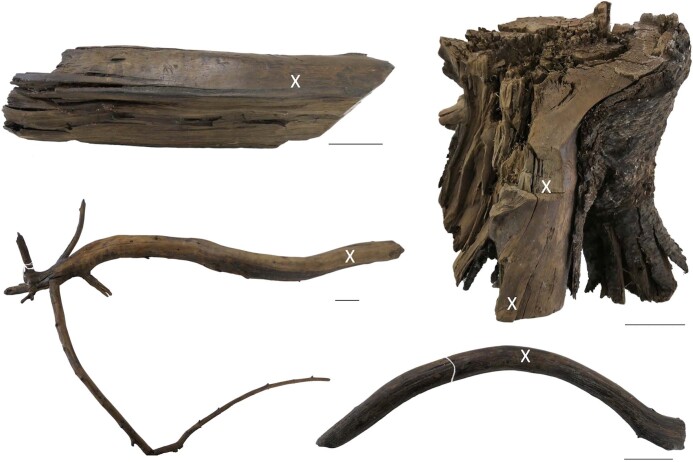
Pictures of the wood items studied. White X indicates the location from where samples were taken. The black bars represent 5 cm. Top left, 4A 59 E3-1; top right, 4A 68 E2-2; bottom left, 4A 68 J4-6; bottom right, 4A 70 B5-14. Photos: M. Kuitems.

**Extended Data Fig. 6:**
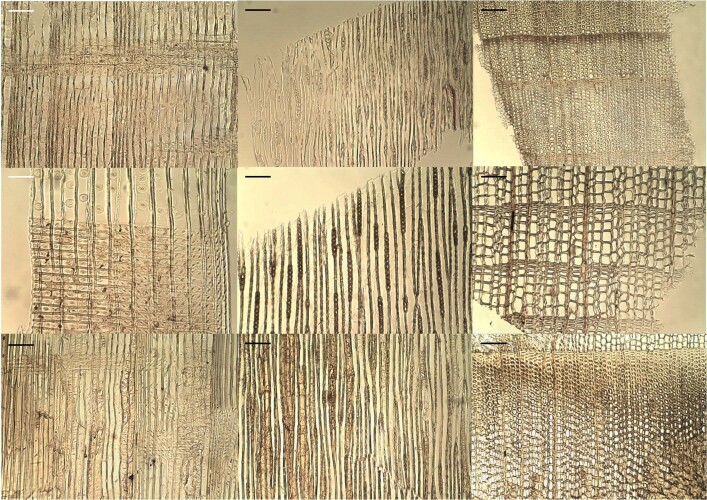
Microscope pictures of the thin slices from the wood samples studied. The white bars represent 0.05 mm, the black bars 0.1 mm. From left to right: radial, tangential and transversal sections of respectively: top, 4A 68 J4-6; middle, 4A 68 E2-2; bottom, 4A 59 E3-1. Photos: M. van Waijjen, BIAX Consult.

Here we present 127 ^14^C measurements, of which 115 were performed at the Centre for Isotope Research (CIO; Groningen), and 12 at the Curt-Engelhorn-Center Archaeometry (CEZA; Mannheim). The samples consisted of 83 individual tree rings from a total of 4 wooden items with find numbers 4A 59 E3-1, 4A 68 E2-2, 4A 68 J4-6 and 4A 70 B5-14 (Extended Data Fig. 5, Supplementary Note 4 and Supplementary Data 2). Unfortunately, the last item is excluded from the remainder of our analysis because it spans only nine years and does not include the ad 993 anomaly and therefore cannot be precisely dated (Supplementary Data 2). Anatomical characteristics such as different numbers of growth rings, varying growth-ring widths and the presence–absence of features such as missing rings show that wood items 4A 59 E3-1, 4A 68 E2-2 and 4A 68 J4-6 come from different trees. Furthermore, they comprise at least two different species, specifically fir, possibly balsam fir (*Abies* cf. *balsamea*), and juniper/thuja (*Juniperus*/*Thuja* type; Extended Data Fig. 6). In addition, the waney edge could be identified in all cases.

The items were found at the locations shown on the site map in Extended Data Fig. 2. The association of these pieces with the Norse is based on detailed research previously conducted by Parks Canada. The determining factors were their location within the Norse deposit and the fact that they had all been modified by metal tools, evident from their characteristically clean, low angle-in cuts^30^. Such implements were not manufactured by the Indigenous inhabitants of the area at the time^30^ (Supplementary Note 4).

Our individual ^14^C results are consistently better than ±2.5‰ (1*σ*), with some averaged results better than ±1.5‰ (about 12 ^14^C yr). Our corpus of replicated measurements is consistent with statistical expectation, and no statistically significant offset (5.1 ± 7.9 ^14^C yr, 1*σ*) was evident between the two ^14^C facilities involved (Supplementary Data 2).

Two steps are used to determine the exact cutting year of each piece of wood. First, the range of possible dates for the waney edges is obtained by standard ^14^C wiggle matching against the Northern Hemisphere calibration curve, IntCal20 (ref. ^23^). Here we use the D_Sequence function in the software OxCal (ref. ^31^) to match the full ^14^C time-series for each item. The resultant 95% probability (2*σ*) ranges for the waney edges all lie between ad 1019 and ad 1024 (Fig. 1c). This indicates that the ad 993 anomaly should be present in each of the wood pieces 26 to 31 years before they were cut. In our numbering system, this corresponds to rings −31 to −26, where the waney edge is assigned to be 0, the penultimate ring is assigned to be −1, and so forth.

**Fig. 2:**
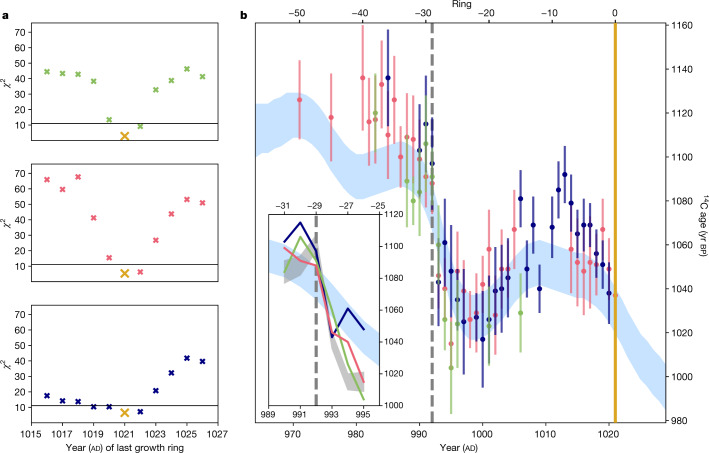
Exact date matches obtained from the *χ*^2^ tests. The wood items are identified as follows: 4A 59 E3-1 (dark green); 4A 68 J4-6 (navy); 4A 68 E2-2 (orange). **a**, Outputs of the *χ*^2^ test against B2018 (ref. ^28^; d.f. = 5, critical value = 11.07, 95% probability), where the gold cross marks the year of best fit for the waney edge. **b**, All of the ^14^C data from 4A 59 E3-1 (*n* = 12, 1*σ*), 4A 68 J4-6 (*n* = 35, 1*σ*) and 4A 68 E2-2 (*n* = 29, 1*σ*) superimposed on IntCal20 (light blue, 1*σ*). Inset: detail of the ^14^C results (error bars omitted for legibility) for growth rings −31 to −26 against B2018 (grey, 1*σ*)^28^ and IntCal20 (light blue). Source data

A second step is then used to determine the exact cutting year of each item. This process hinges on identifying the precise ring in which the ad 993 anomaly is found, and hence the precise date of the waney edge. For this purpose, we use the Classical *χ*^2^ approach^20,32^ to match the ^14^C data from the six rings (−31 to −26) most likely to contain the ad 993 anomaly against a second Northern Hemisphere reference (henceforth B2018)^28^. This dataset is preferred because the ad 993 anomaly is less distinct in the smoothed IntCal20 curve (Fig. 2). The six-ring subsets are compared with B2018 such that *χ*^2^ becomes minimal for the cutting date of each item. The matches are conducted over a range for each waney edge of ad 1016−1026 (Fig. 2a).

**Extended Data Fig. 7:**
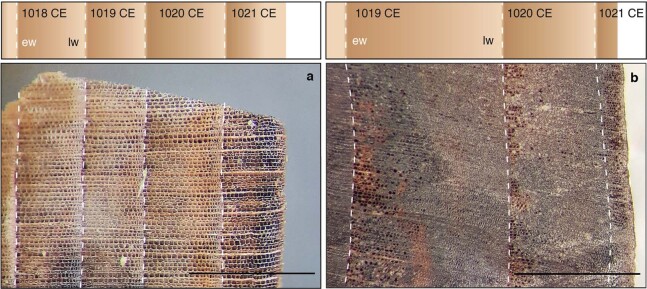
Microscopic depiction of the felling season of the waney edge. The black bars represent 1 mm. ew = early wood, which is formed during the first stage of the growth year; lw = late wood, which is formed at the end of the growth season. **a**, Wood item 4A 68 E2-2; **b**, Wood item 4A 68 J4-6. Photos: P. Doeve.

The optimal *χ*^2^ value for goodness-of-fit for the waney edge in all three cases is ad 1021 (Fig. 2a). While other solutions pass the *χ*^2^ test at 95% probability (ad 1022 for 4A 59 E3-1; ad 1022 for 4A 68 E2-2; ad 1019, ad 1020 and ad 1022 for 4A 68 J4-6), the ideal positioning for the precipitous drop in ^14^C years in each case is when ring −29 corresponds to ad 992 (inset of Fig. 2b). Furthermore, the formation of a small band of earlywood cells in 4A 68 J4-6 indicates a felling season in spring (Extended Data Fig. 7a). The felling season of 4A 68 E2-2 is summer/autumn (Extended Data Fig. 7b). Past polyethylene glycol (Methods) consolidation hinders determination of the felling season of 4A 59 E3-1.

Our result of ad 1021 for the cutting year constitutes the only secure calendar date for the presence of Europeans across the Atlantic before the voyages of Columbus. Moreover, the fact that our results, on three different trees, converge on the same year is notable and unexpected. This coincidence strongly suggests Norse activity at L’Anse aux Meadows in ad 1021. Further evidence reinforces this conclusion. First, the modifications are extremely unlikely to have taken place before this year, because the globally observed sudden decrease in ^14^C values is evident in ring −29. Second, the probability that the items would have been modified at a later stage is also negligible. This is largely because of the fact that they all had their waney edges preserved. This layer would almost certainly have been stripped off during water transport, so the possibility of driftwood is effectively discounted^33^. Further, the Norse would have had no need to reclaim deadwood because fresh wood was abundant in the region at the time^13^. Finally, if it were scavenged material, the probability that all three items would exhibit precisely the same amount of inbuilt age would be vanishingly small.

The Icelandic sagas suggest that the Norse engaged in cultural exchanges with the Indigenous groups of North America^34^. If these encounters indeed occurred, they may have had inadvertent outcomes, such as pathogen transmission^7^, the introduction of foreign flora and fauna species, or even the exchange of human genetic information. Recent data from the Norse Greenlandic population, however, show no evidence of the last of these^8^. It is a matter for future research how the year ad 1021 relates to overall transatlantic activity by the Norse. Nonetheless, our findings provide a chronological anchor for further investigations into the consequences of their westernmost expansion.

## Conclusions

We provide evidence that the Norse were active on the North American continent in the year ad 1021. This date offers a secure juncture for late Viking chronology. More importantly, it acts as a new point-of-reference for European cognisance of the Americas, and the earliest known year by which human migration had encircled the planet. In addition, our research demonstrates the potential of the ad 993 anomaly in atmospheric ^14^C concentrations for pinpointing the ages of past migrations and cultural interactions. Together with other cosmic-ray events, this distinctive feature will allow for the exact dating of many other archaeological and environmental contexts.

## Methods

### Sampling

After careful examination of the transversal and radial sections of the wood, and ring counting, individual samples were collected under a microscope for annual-ring measurement using a steel blade, following the standard procedure for cleaving tree rings. Sample extraction started at the waney edge. For each wood item, the sample of the waney edge was given the number 0, the second-to-last ring was given the number −1, and so forth.

### Sample preparation and measurement

The tree-ring samples were cut into small fragments again using a steel blade. All of the wood samples were chemically pretreated and analysed at CIO, Groningen. For independent control, 12 of the samples were also chemically pretreated and analysed at CEZA, Mannheim. CEZA and CIO recently took part in a multi-laboratory intercomparison exercise to ensure the effectiveness of their pretreatment protocols in which tree-ring samples of unknown age were pretreated to α-cellulose and then analysed for ^14^C concentration by AMS^35^.

### Procedures at CIO, University of Groningen

The first step involves pretreating the samples to α-cellulose, the most rigid and immobile fraction of the wood^36^. The method has previously been described in full^37^. In brief, the wet chemistry involves a series of strong solutions of acid–base–acid and an oxidant, with rinses to neutrality using deionized and ultrapure water after each step. The samples are then either freeze-dried or air-dried at room temperature for 72 h. To eliminate the additive polyethylene glycol (PEG), which was present in all wood items except 4A 68 E2-2, the aqueous pretreatment is preceded by placement of the samples in ultrapure water at 80 °C for 36 h. This latter step builds on past studies of this contaminant^38–40^. In cases where the starting weight was <20 mg, and the wood was not treated with PEG, the holocellulose protocol used at CIO was deemed sufficient^37^ .

Aliquots (about 5 mg, where possible) of the (alpha-)cellulosic product are weighed into tin capsules for combustion in an elemental analyser (IsotopeCube, Elementar). A small amount of the CO_2_(g) released is directed into an isotope ratio mass spectrometer (Isoprime 100) for determination of the stable isotope ratios of C and N, but the majority is cryogenically trapped into Pyrex rigs and reduced to graphite under a stoichiometric excess of H_2_(g) over an Fe(s) catalyst. The graphite (about 2 mg) is subsequently pressed into Al(s) cathodes for measurement by AMS (MICADAS, Ionplus). The data were refined using BATS 4.0 and stored in FileMaker Pro 14.6.0. For quality control purposes, full pretreatment and radioisotope measurements were concurrently conducted on known-age standards (for example, tree-ring material from ad 1503, UK) and background wood (Pleistocene deposit Kitzbühel, Austria). Community-wide isotope ratio mass spectrometry and AMS standards (for example, National Institute of Standards and Technology oxalic acid II, Merck caffeine, and International Atomic Energy Agency C7 and C8) were used to validate the isotope measurements.

### Procedures at CEZA, Mannheim

Samples MAMS-45877–45879 and MAMS-47884–47886 are pretreated as holocellulose and are pretreated using the acid–base–acid method (acid/base/acid, HCl/NaOH/HCl) followed by bleaching with NaClO_2_ to extract the cellulose^41^. The second batch of samples (MAMS-50444–50449) is pretreated as alpha-cellulose following the protocol used by CIO described above. PEG contamination is removed in the same way as at CIO by washing in hot ultrapure water. The cellulose is combusted to CO_2_ in an elemental analyser. CO_2_ is then converted catalytically to graphite. ^14^C is analysed in-house using an AMS instrument of the MICADAS type. The isotopic ratios (^14^C/^12^C of samples, calibration standard oxalic acid II), blanks and control standards are measured simultaneously in the AMS. ^14^C ages are normalized to δ^13^C = −25‰ (ref. ^42^), where δ^13^C = (((^13^C/^12^C)_sample_/(^13^C/^12^C)^standard^)  − 1) × 1,000.

### Models in the program OxCal

All models employ OxCal 4.4 and use its standard Metropolis–Hastings Markov chain Monte Carlo algorithm and default priors^31^. The code for these models is provided in Supplementary Note 5 and in the repository https://github.com/mwdee/LAM1021.

### Averaging

Averages are produced for each sample type using the Sum function in OxCal 4.4. In each case, all of the relevant ^14^C dates are included in bounded phases. The main prior information used by this model is that each date is assumed to be part of a defined group^31^.

### Wiggle matching

^14^C data for each beam are wiggle matched against the IntCal20 calibration curve in OxCal 4.4 using its D_Sequence function^31^. All models show high convergence and run to completion.

### Pattern matching using the *χ*^2^ test

The measured ^14^C concentrations of tree-ring samples are matched to a reference curve through the classical statistical method of the *χ*^2^ test^20,22^, using the following *χ*^2^ function:

$${{X}^{2}}_{(x)}=\mathop{\sum }\limits_{i=1}^{n}\frac{{({R}_{i}-C(x-{r}_{i}))}^{2}}{{\rm{\delta }}{R}_{i}^{2}+{\rm{\delta }}C{(x-{r}_{i})}^{2}}$$

Here *R*_*i*_ ± δ*R*_*i*_ are the measured ^14^C dates of the sample; *C*($x$ − *r*_*i*_) ± δ*C*($x$ − *r*_*i*_) are the ^14^C concentrations of the reference curve for the year ($x$ − *r*_*i*_), where *r*_*i*_ are the tree-ring numbers of the samples analysed; and $x$ is a trial age for the waney edge. Measured dates are matched to the reference data (that is, either higher or lower) in such a way that the *χ*^2^ becomes minimal for a certain value of $x$, which is the best estimate for the felling date of the tree^20^. To match the event accurately, a reference dataset is needed that has single-year resolution. We use B2018 as this reference, which combines many annual ^14^C results for the years relevant to this study^28^. The pattern-matching analyses are predominantly carried out using Python 3 in Jupyter Notebook 6.3.0. The results on each of the wood items studied are shown in Fig. 2.

### Wood taxonomy

From the three main fragments of wood (4A 59 E3-1, 4A 68 E2-2 and 4A 68 J4-6), thin sections are prepared under a stereomicroscope with magnifications of up to 50×. They are cut in three directions (transverse, radial and tangential). As the wood was dry, the sections had to be soaked in soapy water to get rid of air bubbles and to be able to see the diagnostic anatomical features. The slides are examined under a transmitted light microscope with magnifications up to ×400 and identified with the help of relevant literature^43–45^. The three samples do not have any vessels, and therefore must be softwood from conifer species. The most important characteristics for identification are the lack of resin canals, the height of the rays (on average much lower in 4A 68 J4-6 than in the other two samples) and the type, number and distribution of the crossfield pits. Also, presence/absence of axial parenchyma, the shape of the ray cells in crossfields, the pitting in side walls and end walls of the ray cells, and the geographical provenance are taken into account. As wood sample 4A 68 J4-6 is compression wood (reaction wood on the lower side of branches and leaning stems), the distinction between cupressoid and taxoidoid pits cannot be made. The identification for this sample is therefore uncertain with juniper and thuja as possible candidates (*Juniperus*/*Thuja* type). The other two samples are identified with confidence as fir (*Abies*). Within this genus further identification is impossible, but balsam fir (*A. balsamea*), a very common North American species, would be a good match.

### Reporting summary

Further information on research design is available in the Nature Research Reporting Summary linked to this paper.

## Online content

Any methods, additional references, Nature Research reporting summaries, source data, extended data, supplementary information, acknowledgements, peer review information; details of author contributions and competing interests; and statements of data and code availability are available at 10.1038/s41586-021-03972-8.

## Supplementary information

Supplementary Information This file contains Supplementary Notes 1–5 and references. (1) L’Anse aux Meadows; (2) Dating of the site; (3) Length of occupation; (4) Sample materials; (5) Codes.

Reporting Summary

Peer Review File

## Data Availability

All of the data that support the findings of this study are available in the main text or Supplementary Information. Source data are provided with this paper.

## Source data

Source Data Fig. 1
Source Data Fig. 2

## Code availability

The codes of the OxCal models are provided in the Supplementary Information and in the repository https://github.com/mwdee/LAM1021.
