# Supplementary material for: Evidence for European presence in the Americas in ad 1021
Source: Nature. 2021 Oct 20;601(7893):388–91. doi: 10.1038/s41586-021-03972-8 (PMC8770119; doi:10.1038/s41586-021-03972-8)
Supplement: Supplementary file 1 — This file contains Supplementary Notes 1–5 and references. (1) L’Anse aux Meadows; (2) Dating of the site; (3) Length of occupation; (4) Sample materials; (5) Codes. [file 41586_2021_3972_MOESM1_ESM.pdf]

nature portfolio

https://doi.org/10.1038/s41586-021-03972-8

Supplementary information

Evidence for European presence in the Americas in AD 1021

In the format provided by the authors and unedited

Supplementary Information

Evidence for European Presence in the New World in 1021 CE

Margot Kuitems, Birgitta L. Wallace, Charles Lindsay, Andrea Scifo, Petra Doeve, Kevin Jenkins, Susanne Lindauer, Pınar Erdil, Paul M. Ledger, Véronique Forbes, Caroline Vermeeren, Ronny Friedrich & Michael W. Dee

Supplementary Note 1 L'Anse aux Meadows

1.1 Archaeological evidence

In the 1960s, the Norwegian explorer Helge Ingstad and his archaeologist wife Anne Stine Ingstad excavated a genuine Norse site at L'Anse aux Meadows (LAM)[1] and soon LAM received the status of a National Historic Site of Canada. Evidence from subsequent extensive field work by Parks Canada in the mid 1970s linked the remains to the Icelandic Sagas, specifically the Vinland Sagas[2].

Layout of the settlement

The site consists of structures on a dry terrace bordered by peat bogs. The area west of the site is dominated by wet sedge peat, while the inland side is characterised by a *Sphagnum* bog. The structures involve three dwelling complexes spaced at even distances east of a small brook. On the west side of the brook a furnace is situated for iron production from local bog ore. Each dwelling complex comprises a large hall and one or two small buildings (Extended Data Fig. 2)[3,4].

Palynological analysis suggests that the vegetation at the site in the Viking Age was more or less similar to the present day. The area consisted of heathland, grasses, sedges and a few trees

such as balsam fir, willow, alder. A dense forest of balsam fir and spruce existed close to the site. Today's forest line is more than 15 km away due to recent deforestation[5]. During the Medieval Warm Period (~ 950-1250 CE) LAM probably remained snow free[6].

Architecture

The halls (indicated by A, D, and F in Extended Data Fig. 2) were large and firmly built. They had 1.5 to 2m-thick walls and interior posts to support a permanent, sod roof. The walls were made of sod over a timber frame.

Function and social organisation

The solid roofs at LAM protected the inhabitants from the winter and indicate that the site was intended to be occupied year-round[7]. The halls had large storage rooms where goods were collected, presumably for shipment to Greenland[3]. Wood fragments and other plant remains such as butternut (*Juglans cinerea*) found much further south in the Americas, and certainly not indigenous to Newfoundland, have been found at LAM[3,8]. Further, each hall had rooms for specific activities such as iron forging and carpentry. The size of the halls was so large that only high-status people could afford them, whereas the smaller buildings (indicated by B, C, E, and G in Extended Data Fig. 2) were of the kind used by the rest of the group. The lack of a byre which are so prominent on Icelandic and Greenland farms suggests that livestock were not present in large numbers. Altogether the buildings could accommodate 70-90 people[9]. The primary function of the settlement is presumed to have been a gateway between Greenland and areas to the south, likely including the lands around the Gulf of St. Lawrence[4].

Artefacts and wooden remains

More than 2000 well-preserved items were found in the bog deposited in three stratigraphic layers. The Norse stratum was in the middle, and yielded wood remains that were worked by metal tools, whereas the layers below and above contained wood solely worked with indigenous stone tools[4,8].

Metal remains that have been found here and there at the site include a bronze cloak pin, discarded nails and rivets, and smelting and smithing slag[8,10.]

1.2 Historical evidence

Icelandic literature from the 13th century is an invaluable source of information about Norse history. In particular, the Vinland Sagas describe Norse voyages from their settlements in Greenland to a 'new world', which has long been interpreted to be the Americas. The Vinland Sagas exist in two versions, namely the Saga of the Greenlanders (*Grœnlendinga saga*) and Saga of Erik the Red (*Eiríks saga rauða*), which convey the same events but differ considerably in detail. Both Sagas tell of exploring new lands and after a short, but undisclosed period of time, culminate with the return to Greenland[11].

The Saga of Erik the Red reports an expedition to the Vinland previously discovered accidentally by the Norseman, Leif Eriksson, son of Greenlandic chief, Erik the Red. The expedition was led by the Icelandic aristocrat, Thorfinn Karlsefni, and his wife, Gudrid, Leif Eriksson's widowed sister-in-law[11].

The Saga of the Greenlanders describes four expeditions to the Vinland following the inadvertent discovery of lands by the Norseman Bjarni Herjolfsson. The first of these

expeditions is led by Leif Eriksson. The following expeditions were led by his family members, including Thorfinn Karlsefni[11].

In both Sagas, the newly discovered world is described as three separate lands, namely *Vinland* (Wine land), *Markland* (Forest land) and *Helluland* (Land of stone slabs). Of these, Vinland is believed to be what is known today as Newfoundland and the coasts around the Gulf of St. Lawrence[8,11].

The Saga of Erik the Red pictures a settlement called *Straumfjörðr* (Straumfjord, 'Fjord of Currents') near an island surrounded by strong currents. Straumfjord matches the characteristics of the settlement at LAM very well. It is described as a winter base from which expeditions were launched in summer, including south to *Hóp*, located in a tidal lagoon rich in resources which were gathered and brought back to Straumfjord[8,11].

The Saga of the Greenlanders tells about one settlement called *Leifsbúðir* (Leif's Camp)[11]. It is described as somewhat of a mixture between Straumfjord and Hóp, functioning as both a camp for winter and for exploiting summer resources[8], but alternatively others have interpreted Leif's Camp, Straumfjord and Hóp as three separate locations[12-14].

Supplementary Note 2 Dating of the site

2.1 Archaeological evidence

The architectural style, the construction materials, and a handful of artefacts in the buildings are similar to Icelandic-Early Greenland Style of the 11th century[3,8]. Furnaces for smelting iron (as indicated by J in Extended Data Fig. 2) were not common but some are known on Icelandic farms[7].

2.2 Previously published radiocarbon dates

55 ^{14}C dates on the site relate to the Norse occupation. They range between 600–1200 CE (95% probability, Source Data Fig. 1; Extended Fig. 3).

Supplementary Note 3 Length of occupation

3.1 Archaeological evidence

Archaeological evidence from LAM is unequivocal that the Norse occupation lasted a brief period[10]. The main evidence for this is the absence of a burial ground, and the diminutive amount of garbage and organic accumulation proximal to the site. This interpretation is supported by comparisons with the refuse excavated at Norse sites on Iceland and Greenland. Moreover, the buildings at LAM do not show any sign of refurbishment or renewal.

3.2 Historical evidence

The Vinland Sagas are explicit that all expeditionary groups ultimately returned to Greenland[11]. Based on information in the Sagas[11], one can make an estimate of the minimum duration of the occupation of LAM. The reconstructed time between the first arrival of the Norse and their last-mentioned visit is schematically shown in Extended Data Fig. 4.

Supplementary Note 4 Sample Materials

Timber was a key building material for the Vikings[15], and in the Viking Age, balsam fir and spruce grew abundantly in the close vicinity of LAM[5]. Accordingly, many wooden remains have been recovered from the site. In this study, four wooden objects Extended Data Fig. 5 from different locations at LAM (Extended Data Fig. 2) were sampled for high-precision ^{14}C measurement. The wooden objects were all identified as Norse.

Wood item 4A 59 E3-1 is a log from balsam fir (*Abies* cf. *balsamea*) found in the western slope of a gravel bank. It is about 0.5 metres in length and shows clear and sharp cuts on both ends and cuts along the length that have been made by a flat low-angle metal tool. Wood item 4A 68 E2-2 is a tree stump of fir (*Abies* species). The stems of the tree are cut off by quick slashes with a metal tool. The tool marks were described by Gleeson (Parks Canada report 1979, p8) as depicting the "distinctive shearing and well-defined ridging of a metal flat, low angle blade edge". ^{14}C measurement performed in 1985 dates this piece (TO-119) to 896–1114 CE (95% probability).

Wood item 4A 68 J4-6 is a branch that could not be identified at genus level. It is possibly juniper/thuja (Juniperus/Thuja-type). The branch is more than a metre in length. It was cut off from the tree stem and hence shows cut marks at its base. Smaller branches project from the main stem. Wood item 4A 70 B5-14 is a branch that has not been taxonomically investigated. It was located within the peat. It has clear cut marks on one end.

Supplementary Note 5 Codes

5.1 OxCal codes for summarising the age ranges of legacy ^{14}C dates by sample type

Averages are produced for each sample type using the Sum function in OxCal. In each case, all the relevant results are included in Bounded Phases.

```
Options()
 {
  Resolution=1;
 };
 Plot()
 {
  Outlier_Model("General",T(5),U(0,4),"t");
  Sequence("Outer Tree-rings")
  {
   Boundary("Start Outer Tree-rings");
   Phase("Outer Tree-rings Phase")
   {
    R_Date("TO-116", 1440, 50)
    {
     Outlier(0.05);
     color="DarkGoldenRod";
    };
    R_Date("TO-117", 1030, 50)
    {
     Outlier(0.05);
     color="DarkGoldenRod";
    };
    R_Date("TO-118", 990, 30)
    {
     Outlier(0.05);
     color="DarkGoldenRod";
    };
    R_Date("TO-119", 1040, 30)
    {
     Outlier(0.05);
     color="DarkGoldenRod";
    };
    Sum("Outer Tree-rings (Average)")
    {
     color="DarkGoldenRod";
    };
   };
   Boundary("End Outer Tree-rings");
  };
  Sequence("Sod")
  {
   Boundary("Start Sod");
   Phase("Sod Phase")
   {
    R_Date("T-530", 950, 90)
    {
     Outlier(0.05);
     color="YellowGreen";
    };
    R_Date("T-531", 950, 50)
    {
     Outlier(0.05);
     color="YellowGreen";
    };
    R_Date("T-817", 1300, 70)
    {
     Outlier(0.05);
     color="YellowGreen";
    };
    R_Date("T-818", 1320, 80)
    {
     Outlier(0.05);
     color="YellowGreen";
    };
```

```
  Sum("Sod (Average)")
  {
   color="YellowGreen";
  };
 };
 Boundary("End Sod");
};
R_Date("Bone (St-2665)", 1085, 110)
{
 Outlier(0.05);
 color="LightBlue";
};
Sequence("Wood")
{
 Boundary("Start Wood");
 Phase("Wood Phase")
 {
  R_Date("S-1092", 1410, 90)
  {
   Outlier(0.05);
   color="Red";
  };
  R_Date("S-1346", 1330, 80)
  {
   Outlier(0.05);
   color="Red";
  };
  R_Date("S-1109", 1305, 60)
  {
   Outlier(0.05);
   color="Red";
  };
  R_Date("S-1343", 1250, 70)
  {
   Outlier(0.05);
   color="Red";
  };
  R_Date("S-1090", 1230, 70)
  {
   Outlier(0.05);
   color="Red";
  };
  R_Date("S-1118", 1210, 100)
  {
   Outlier(0.05);
   color="Red";
  };
  R_Date("S-1357", 1160, 60)
  {
   Outlier(0.05);
   color="Red";
  };
  R_Date("S-1120", 1095, 100)
  {
   Outlier(0.05);
   color="Red";
  };
  R_Date("S-1110", 1090, 60)
  {
   Outlier(0.05);
   color="Red";
  };
  R_Date("S-1093", 1070, 65)
  {
   Outlier(0.05);
   color="Red";
  };
  R_Date("S-1113", 1040, 50)
  {
   Outlier(0.05);
   color="Red";
  };
  R_Date("S-1111", 960, 105)
  {
   Outlier(0.05);
```

```
   color="Red";
  };
  R_Date("S-1091", 865, 65)
  {
   Outlier(0.05);
   color="Red";
  };
  R_Date("S-1340", 1050, 65)
  {
   Outlier(0.05);
   color="Red";
  };
  R_Date("S-1355", 955, 100)
  {
   Outlier(0.05);
   color="Red";
  };
  R_Date("Qu-350", 1400, 80)
  {
   Outlier(0.05);
   color="Red";
  };
  R_Date("WAT-436", 1080, 40)
  {
   Outlier(0.05);
   color="Red";
  };
  Sum("Wood (Average)")
  {
   color="Red";
  };
 };
 Boundary("End Wood");
};
Sequence("Burnt Wood")
{
 Boundary("Start Burnt Wood");
 Phase("Burnt Wood Phase")
 {
  R_Date("S-1101", 1075, 60)
  {
   Outlier(0.05);
   color="DarkBrown";
  };
  R_Date("S-1119", 1375, 115)
  {
   Outlier(0.05);
   color="DarkBrown";
  };
  R_Date("S-1358", 1345, 65)
  {
   Outlier(0.05);
   color="DarkBrown";
  };
  R_Date("S-1115", 1210, 45)
  {
   Outlier(0.05);
   color="DarkBrown";
  };
  R_Date("S-1342", 1200, 100)
  {
   Outlier(0.05);
   color="DarkBrown";
  };
  R_Date("S-1114", 1120, 120)
  {
   Outlier(0.05);
   color="DarkBrown0";
  };
  R_Date("S-1112", 1105, 45)
  {
   Outlier(0.05);
   color="DarkBrown";
  };
  Sum("Burnt Wood (Average)")
```

```
   {
   color="DarkBrown";
  };
 };
 Boundary("End Burnt Wood");
};
Sequence("Charcoal")
{
 Boundary("Start Charcoal");
 Phase("Charcoal Phase")
 {
  R_Date("T-310", 1310, 130)
  {
   Outlier(0.05);
   color="Black";
  };
  R_Date("T-306", 1210, 110)
  {
   Outlier(0.05);
   color="Black";
  };
  R_Date("T-309", 1240, 130)
  {
   Outlier(0.05);
   color="Black";
  };
  R_Date("T-364", 1050, 70)
  {
   Outlier(0.05);
   color="Black";
  };
  R_Date("T-324", 1130, 70)
  {
   Outlier(0.05);
   color="Black";
  };
  R_Date("T-325", 1080, 70)
  {
   Outlier(0.05);
   color="Black";
  };
  R_Date("T-326", 1250, 70)
  {
   Outlier(0.05);
   color="Black";
  };
  R_Date("T-327", 870, 70)
  {
   Outlier(0.05);
   color="Black";
  };
  R_Date("T-366", 1090, 90)
  {
   Outlier(0.05);
   color="Black";
  };
  R_Date("T-393", 890, 70)
  {
   Outlier(0.05);
   color="Black";
  };
  R_Date("T-367", 1130, 70)
  {
   Outlier(0.05);
   color="Black";
  };
  R_Date("S-1108", 1300, 110)
  {
   Outlier(0.05);
   color="Black";
  };
  R_Date("S-1350", 1320, 60)
  {
   Outlier(0.05);
   color="Black";
```

```
    };
    R_Date("S-1102", 1130, 115)
    {
     Outlier(0.05);
     color="Black";
    };
    R_Date("S-1339", 1330, 105)
    {
     Outlier(0.05);
     color="Black";
    };
    R_Date("S-1348", 1235, 65)
    {
     Outlier(0.05);
     color="Black";
    };
    R_Date("S-1347", 1195, 60)
    {
     Outlier(0.05);
     color="Black";
    };
    R_Date("S-1106", 1185, 110)
    {
     Outlier(0.05);
     color="Black";
    };
    R_Date("S-1352", 1160, 65)
    {
     Outlier(0.05);
     color="Black";
    };
    R_Date("S-1127", 1185, 105)
    {
     Outlier(0.05);
     color="Black";
    };
    R_Date("S-1351", 1300, 60)
    {
     Outlier(0.05);
     color="Black";
    };
    R_Date("Beta-292106", 1150, 30)
    {
     Outlier(0.05);
     color="Black";
    };
    Sum("Charcoal (Average)")
    {
     color="Black";
    };
   };
   Boundary("End Charcoal");
  };
 };
```

5.2 OxCal codes for establishing the felling dates of the individual samples

4A 59 E3-1

```
Options()
 {
  Resolution=1;
 };
 Plot()
 {
  Outlier_Model("General",T(5),U(0,4),"t");
  D_Sequence(" 4A 59 E3-1")
  {
   R_Date("GrM-25519",1120,18)
   {
    Outlier(0.05);
   };
   Gap(5);
   R_Date("GrM-25135",1089,21)
   {
    Outlier(0.05);
   };
   Gap(1);
   R_Combine("GrM-25134, 25516 & 25517")
   {
    Outlier(0.05);
    R_Date("GrM-25134",1036,21);
    R_Date("GrM-25516",1097,18);
    R_Date("GrM-25517",1095,18);
   };
   Gap(1);
   R_Date("GrM-25133",1084,20)
   {
    Outlier(0.05);
   };
   Gap(1);
   R_Date("GrM-25131",1106,22)
   {
    Outlier(0.05);
   };
   Gap(1);
   R_Combine("GrM-25130 & 25515")
   {
    Outlier(0.05);
    R_Date("GrM-25130",1081,20);
    R_Date("GrM-25130",1099,18);
   };
   Gap(1);
   R_Date("GrM-25523",1060,18)
   {
    Outlier(0.05);
   };
   Gap(1);
   R_Combine("GrM-25128 & 25513")
   {
    Outlier(0.05);
    R_Date("GrM-25128",1011,21);
    R_Date("GrM-25513",1037,18);
   };
   Gap(1);
   R_Date("GrM-25138",1004,21)
   {
    Outlier(0.05);
   };
   Gap(1);
   R_Date("GrM-25136",1024,20)
   {
    Outlier(0.05);
   };
   Gap(5);
   R_Date("GrM-25518",1023,18)
   {
    Outlier(0.05);
   };
```

```
  Gap(5);
  R_Date("GrM-25520",1029,18)
  {
   Outlier(0.05);
  };
  Gap(15);
  Date("Felling Date 4A 59 E3-1")
  {
   color="Forestgreen";
  };
 };
};
```

4A 68 E2-2

```
Options()
 {
 Resolution=1;
 };
 Plot()
 {
 Outlier_Model("General",T(5),U(0,4),"t");
 D_Sequence("4A 68 E2-2")
  {
  R_Date("GrM-24845",1126,18)
  {
   Outlier(0.05);
  };
  Gap(5);
  R_Date("GrM-24844",1118,20)
  {
   Outlier(0.05);
  };
  Gap(5);
  R_Date("GrM-24843",1136,24)
  {
   Outlier(0.05);
  };
  Gap(1);
  R_Date("GrM-24847",1116,20)
  {
   Outlier(0.05);
  };
  Gap(1);
  R_Date("GrM-24846",1117,20)
  {
   Outlier(0.05);
  };
  Gap(1);
  R_Date("GrM-24842",1133,20)
  {
   Outlier(0.05);
  };
  Gap(1);
  R_Date("GrM-24841",1110,20)
  {
   Outlier(0.05);
  };
  Gap(1);
  R_Date("GrM-24840",1126,20)
  {
   Outlier(0.05);
  };
  Gap(1);
  R_Combine("GrM-24839 & MAMS-50444")
  {
   Outlier(0.05);
   R_Date("GrM-24839",1108,18);
   R_Date("MAMS-50444",1090,21);
  };
  Gap(1);
  R_Date("GrM-24838",1109,20)
  {
   Outlier(0.05);
```

```
};
Gap(1);
R_Date("GrM-24835",1108,20)
{
 Outlier(0.05);
};
Gap(1);
R_Date("GrM-24834",1099,18)
{
 Outlier(0.05);
};
Gap(1);
R_Combine("GrM-24833 & MAMS-50445")
{
 Outlier(0.05);
 R_Date("GrM-24833",1099,18);
 R_Date("MAMS-50445",1081,21);
};
Gap(1);
R_Combine("GrM-24832 & MAMS-50446")
{
 Outlier(0.05);
 R_Date("GrM-24832",1101,18);
 R_Date("MAMS-50446",1071,21);
};
Gap(1);
R_Combine("GrM-24831 & MAMS-50447")
{
 Outlier(0.05);
 R_Date("GrM-24831",1058,20);
 R_Date("MAMS-50447",1031,22);
};
Gap(1);
R_Combine("GrM-24830 & MAMS-50448")
{
 Outlier(0.05);
 R_Date("GrM-24830",1041,20);
 R_Date("MAMS-50448",1038,21);
};
Gap(1);
R_Combine("GrM-22470, GrM-24827 & MAMS-50449")
{
 Outlier(0.05);
 R_Date("GrM-22470",1008,18);
 R_Date("GrM-24827",1026,21);
 R_Date("MAMS-50449",1013,21);
};
Gap(1);
R_Date("GrM-24828",1048,18)
{
 Outlier(0.05);
};
Gap(1);
R_Combine("GrM-22469 & GrM-24826")
{
 Outlier(0.05);
 R_Date("GrM-22469",1034,18);
 R_Date("GrM-24826",1048,24);
};
Gap(1);
R_Combine("GrM-22467 & GrM-22468")
{
 Outlier(0.05);
 R_Date("GrM-22467",1033,18);
 R_Date("GrM-22468",1018,18);
};
Gap(1);
R_Date("GrM-22466",1029,18)
{
 Outlier(0.05);
};
Gap(1);
R_Combine("GrM-22462 & GrM-22465")
{
 Outlier(0.05);
```

```
 R_Date("GrM-22462",1034,18);
 R_Date("GrM-22465",1049,18);
};
Gap(1);
R_Date("GrM-22461",1058,18)
{
 Outlier(0.05);
};
Gap(1);
R_Date("GrM-22460",1028,18)
{
 Outlier(0.05);
};
Gap(1);
R_Date("GrM-22459",1049,18)
{
 Outlier(0.05);
};
Gap(1);
R_Combine("GrM-22457 & GrM-22458")
{
 Outlier(0.05);
 R_Date("GrM-22457",1073,18);
 R_Date("GrM-22458",1025,18);
};
Gap(1);
R_Date("GrM-22455",1067,18)
{
 Outlier(0.05);
};
Gap(9);
R_Date("GrM-21812",1058,20)
{
 Outlier(0.05);
};
Gap(1);
R_Date("GrM-21811",1052,20)
{
 Outlier(0.05);
};
Gap(1);
R_Date("GrM-21810",1048,20)
{
 Outlier(0.05);
};
Gap(1);
R_Date("GrM-21809",1052,21)
{
 Outlier(0.05);
};
Gap(1);
R_Combine("GrM-21808 & MAMS-45877")
{
 Outlier(0.05);
 R_Date("GrM-21808",1055,21);
 R_Date("MAMS-45877",1048,20);
};
Gap(1);
R_Combine("GrM-21807 & MAMS-45879")
{
 Outlier(0.05);
 R_Date("GrM-21807",1062,20);
 R_Date("MAMS-45879",1072,21);
};
Gap(1);
R_Combine("GrM-21806 & MAMS-45878")
{
 Outlier(0.05);
 R_Date("GrM-21806",1036,20);
 R_Date("MAMS-45878",1064,21);
};
Gap(1);
R_Date("GrM-21805 (Felling Date)",1037,22)
{
 color="Orangered";
```

```
   Outlier(0.05);
  };
 };
};
```

4A 68 J4-6

(To run without Outlier Analysis, samples “GrM-18960 & GrM-21799” and “GrM-18966 & GrM-21600” need to be excluded to achieve sufficient Agreement but the results are identical)

```
Options()
 {
  Resolution=1;
 };
 Plot()
 {
  Outlier_Model("General",T(5),U(0,4),"t");
  D_Sequence("4A 68 J4-6")
  {
   R_Date("GrM-25151",1136,22)
   {
    Outlier(0.05);
   };
   Gap(5);
   R_Date("GrM-25146",1103,21)
   {
    Outlier(0.05);
   };
   Gap(1);
   R_Date("GrM-25145",1115,22)
   {
    Outlier(0.05);
   };
   Gap(1);
   R_Date("GrM-25143",1097,21)
   {
    Outlier(0.05);
   };
   Gap(1);
   R_Date("GrM-25144",1043,20)
   {
    Outlier(0.05);
   };
   Gap(1);
   R_Date("GrM-25142",1061,20)
   {
    Outlier(0.05);
   };
   Gap(1);
   R_Date("GrM-25141",1048,21)
   {
    Outlier(0.05);
   };
   Gap(1);
   R_Combine("GrM-21612 & GrM-25031")
   {
    Outlier(0.05);
    R_Date("GrM-21612",1036,20);
    R_Date("GrM-25031",1033,21);
   };
   Gap(1);
   R_Date("GrM-21611",1025,24)
   {
    Outlier(0.05);
   };
   Gap(2);
   R_Combine("GrM-21610, GrM-25510 & GrM-25511")
   {
    Outlier(0.05);
    R_Date("GrM-21610",999,20);
    R_Date("GrM-25510",1039,18);
    R_Date("GrM-25511",1040,18);
```

```
};
Gap(1);
R_Date("GrM-21609",1017,22)
{
 Outlier(0.05);
};
Gap(1);
R_Date("GrM-21608",1026,20)
{
 Outlier(0.05);
};
Gap(1);
R_Date("GrM-21607",1039,20)
{
 Outlier(0.05);
};
Gap(1);
R_Date("GrM-21606",1040,20)
{
 Outlier(0.05);
};
Gap(1);
R_Date("GrM-21605",1045,18)
{
 Outlier(0.05);
};
Gap(2);
R_Combine("GrM-18966 & GrM-21600")
{
 Outlier(0.05);
 R_Date("GrM-18966",1094,18);
 R_Date("GrM-21600",1067,20);
};
Gap(1);
R_Combine("GrM-18623 & GrM-21596")
{
 Outlier(0.05);
 R_Date("GrM-18623",1058,18);
 R_Date("GrM-21596",1040,18);
};
Gap(1);
R_Combine("GrM-18965 & GrM-21599")
{
 Outlier(0.05);
 R_Date("GrM-18965",1091,18);
 R_Date("GrM-21599",1044,20);
};
Gap(1);
R_Combine("GrM-18622, GrM-21595 & MAMS-47884")
{
 Outlier(0.05);
 R_Date("GrM-18622",1057,18);
 R_Date("GrM-21595",1037,18);
 R_Date("MAMS-47884",1023,20);
};
Gap(2);
R_Combine("GrM-18962 & GrM-21597")
{
 Outlier(0.05);
 R_Date("GrM-18962",1086,18);
 R_Date("GrM-21597",1045,21);
};
Gap(1);
R_Combine("GrM-18961 & GrM-21800")
{
 Outlier(0.05);
 R_Date("GrM-18961",1092,18);
 R_Date("GrM-21800",1076,20);
};
Gap(1);
R_Combine("GrM-18960 & GrM-21799")
{
 Outlier(0.05);
 R_Date("GrM-18960",1104,18);
 R_Date("GrM-21799",1078,20);
```

```
  };
  Gap(1);
  R_Combine("GrM-18957 & GrM-21798")
  {
   Outlier(0.05);
   R_Date("GrM-18957",1091,18);
   R_Date("GrM-21798",1066,20);
  };
  Gap(1);
  R_Combine("GrM-18621, GrM-21593 & GrM-21794")
  {
   Outlier(0.05);
   R_Date("GrM-18621",1065,18);
   R_Date("GrM-21593",1060,20);
   R_Date("GrM-21794",1070,20);
  };
  Gap(1);
  R_Combine("GrM-18955, GrM-21797 & MAMS-47885")
  {
   Outlier(0.05);
   R_Date("GrM-18955",1075,18);
   R_Date("GrM-21797",1056,22);
   R_Date("MAMS-47885",1073,20);
  };
  Gap(1);
  R_Combine("GrM-21793, GrM-18618, GrM-18620 & MAMS-47886")
  {
   Outlier(0.05);
   R_Date("GrM-21793",1055,20);
   R_Date("GrM-18618",1068,18);
   R_Date("GrM-18620",1069,18);
   R_Date("MAMS-47886", 1093, 25);
  };
  Gap(1);
  R_Combine("GrM-21792, GrM-18616 & GrM-18617")
  {
   Outlier(0.05);
   R_Date("GrM-21792",1051,20);
   R_Date("GrM-18616",1046,18);
   R_Date("GrM-18617",1071,18);
  };
  Gap(1);
  R_Combine("GrM-21791, GrM-18614 & GrM-18615")
  {
   Outlier(0.05);
   R_Date("GrM-21791",1041,20);
   R_Date("GrM-18614",1055,18);
   R_Date("GrM-18615",1055,18);
  };
  Gap(1);
  R_Combine("GrM-18954 & GrM-21796")
  {
   Outlier(0.05);
   R_Date("GrM-18954",1038,18);
   R_Date("GrM-21796",1039,21);
  };
  Gap(1);
  Date("Felling Date 4A 68 J4-6")
  {
   color="Navy";
  };
 };
};
```

Supplementary Note 6 References

1. Ingstad, A. S. & Ingstad, H. *The Norse Discovery of America.* Vols. I and II. (University of Oslo Press, 1986).
2. Lindsay, C. S. Was L'Anse aux Meadows a Norse Outpost? *Canadian Geographic Journal* **94**, 36–43 (1977).
3. Wallace, B. L. L'Anse aux Meadows and Vinland: an abandoned experiment. in: *Contact, Continuity, and Collapse : the Norse Colonization of the North Atlantic* (ed. Barrett, J.) 207–238 (Brepols, 2003).
4. Wallace, B. L. L'Anse aux Meadows: Different Disciplines, Divergent Views. in *Viking Settlements & Viking Society*, *Papers from the Proceedings of the Sixteenth Viking Congress, Reykjavík and Reykholt, 16-23 August 2009* (eds. Sigmundsson, S. et al.) 448-468. (Reykjavik: Hið Íslenzka fornleifafélag & University of Iceland Press, 2011).
5. Davis, A. M., McAndrews, J. H. & Wallace, B. L. Paleoenvironment and the archaeological record at the L'Anse aux Meadows Site, Newfoundland. *Geoarchaeology* **3**, 53–64 (1988).
6. Ogilvie, A. E., Barlow, L. K. & Jennings, A. E. North Atlantic climate c. AD 1000: Millennial reflections on the Viking discoveries of Iceland, Greenland and North America. *Weather* **55**, 34–45 (2000).
7. Wallace, B. L. L'Anse Aux Meadows, Leif Eriksson's Home in Vinland. *J. North Atl.* **2**, 114–125 (2009).
8. Wallace, B. L. The Norse in Newfoundland: L'Anse aux Meadows and Vinland. *Newfoundland and Labrador Studies* **19,** (2003).
9. Wallace, B. L. Westward Vikings: The Saga of L'Anse aux Meadows. St John's, Newfoundland: Historic Sites Association of Newfoundland and Labrador, 2006).

10. Wallace, B. L. L'Anse aux Meadows National Historic Site. in: *Archaeology in America: An Encyclopedia* (eds. Cordell, L. S., Lightfoot, K., McManamon, F. & Milner, G.) 78–83 (ABC-CLIO, 2009).
11. Smiley, J. *The Sagas of the Icelanders.* (Penguin, 2005).
12. Þórðarson, M. The Vinland Voyages. *American Geographical Society* **14.** (1930).
13. Bergthórsson, P. *The Wineland Millennium Saga and Evidence*. Reykjavik: Mál og menning. (2000).
14. Sigurðsson, G. *The Medieval Icelandic Saga and Oral Tradition. A Discourse on Method.* Publications of the Milman Parry Collection of Oral Literature No. 2. Translated by Nicholas Jones. Cambridge, Massachusetts: The Milman Parry Collection of Oral Literature, Harvard University (2004).
15. Pinta, E. Norse management of wooden resources across the north Atlantic: Highlights from the Norse Greenlandic settlements. *Environmental Archaeology* **26**, 1-13 (2018).
