# Supplementary material for: Evidence for European presence in the Americas in ad 1021
Source: Nature. 2021 Oct 20;601(7893):388–91. doi: 10.1038/s41586-021-03972-8 (PMC8770119; doi:10.1038/s41586-021-03972-8)
Supplement: Supplementary file 2 — Reporting Summary [file 41586_2021_3972_MOESM2_ESM.pdf]

nature portfolio

Corresponding author(s): Michael Dee
Margot Kuitems

Last updated by author(s): Aug 20, 2021

Reporting Summary

Nature Portfolio wishes to improve the reproducibility of the work that we publish. This form provides structure for consistency and transparency in reporting. For further information on Nature Portfolio policies, see our Editorial Policies and the Editorial Policy Checklist.

Statistics

For all statistical analyses, confirm that the following items are present in the figure legend, table legend, main text, or Methods section.

n/a	Confirmed	
☐	☒	The exact sample size (*n*) for each experimental group/condition, given as a discrete number and unit of measurement
☐	☒	A statement on whether measurements were taken from distinct samples or whether the same sample was measured repeatedly
☐	☒	The statistical test(s) used AND whether they are one- or two-sided<br>*Only common tests should be described solely by name; describe more complex techniques in the Methods section.*
☒	☐	A description of all covariates tested
☐	☒	A description of any assumptions or corrections, such as tests of normality and adjustment for multiple comparisons
☐	☒	A full description of the statistical parameters including central tendency (e.g. means) or other basic estimates (e.g. regression coefficient) AND variation (e.g. standard deviation) or associated estimates of uncertainty (e.g. confidence intervals)
☒	☐	For null hypothesis testing, the test statistic (e.g. *F*, *t*, *r*) with confidence intervals, effect sizes, degrees of freedom and *P* value noted<br>*Give P values as exact values whenever suitable.*
☐	☒	For Bayesian analysis, information on the choice of priors and Markov chain Monte Carlo settings
☒	☐	For hierarchical and complex designs, identification of the appropriate level for tests and full reporting of outcomes
☒	☐	Estimates of effect sizes (e.g. Cohen's *d*, Pearson's *r*), indicating how they were calculated

Our web collection on statistics for biologists contains articles on many of the points above.

Software and code

Policy information about availability of computer code

Data collection	Data were collected by authors from Canada (Government of Canada, Parks Canada Agency), Netherlands (University of Groningen, BIAX Consult, Laboratory for Dendrochronology at BAAC and Rijksdienst voor het Cultureel Erfgoed) and Germany (CEZA Mannheim) using the software below. Radiocarbon dates were acquired on a MICADAS AMS system using BATS software 4.0<br>Raw data is stored in FileMaker Pro 14.6.0
Data analysis	OxCal 4.4 using standard Metropolis-Hastings Markov Chain Monte Carlo (MCMC) algorithm and default priors<br>Canadian Archaeological Radiocarbon Database 2.0<br>Python 3 in Jupyter Notebook 6.3.0<br>Inkscape 1.0.1<br>Further details on the methods and relevant codes are included in the manuscript and the Supplementary Information and are available on this repository: https://github.com/mwdee/LAM1021

For manuscripts utilizing custom algorithms or software that are central to the research but not yet described in published literature, software must be made available to editors and reviewers. We strongly encourage code deposition in a community repository (e.g. GitHub). See the Nature Portfolio guidelines for submitting code & software for further information.

Data

Policy information about availability of data

All manuscripts must include a data availability statement. This statement should provide the following information, where applicable:

- Accession codes, unique identifiers, or web links for publicly available datasets
- A description of any restrictions on data availability
- For clinical datasets or third party data, please ensure that the statement adheres to our policy

All the data that support the findings of this study are available in the main text, Extended Data files or Supplementary Information files.

Field-specific reporting

Please select the one below that is the best fit for your research. If you are not sure, read the appropriate sections before making your selection.

☒ Life sciences ☐ Behavioural & social sciences ☐ Ecological, evolutionary & environmental sciences

For a reference copy of the document with all sections, see nature.com/documents/nr-reporting-summary-flat.pdf

Life sciences study design

All studies must disclose on these points even when the disclosure is negative.

Sample size	We did not rely on statistical methods to predetermine sample sizes. Sample sizes in this study on an archaeological site were limited by the availability of excavated wood specimens and numbers of growth rings they contained.
Data exclusions	Data was obtained on sample 4A 70 B5-14 (see Source Data Fig. 2). However, no pattern matching was attempted for this sample, because the 993 CE anomaly was clearly not evident and therefore it could not be precisely dated. Two sample measurements that failed the community's standard chi square statistical test for congruity were excluded from analysis (see below).<br>Further details are provided in the manuscript and Extended Data.
Replication	34 samples were pretreated and radiocarbon dated more than once (as duplicates, triplicates and one quadruplicate). 32 successfully passed the community's standard (chi square) statistical test for congruity (94.1 vs 95.4% expected probability). On 12 occasions, the repeated pretreatments and measurements were conducted by another radiocarbon facility (CEZA Mannheim). We concentrated the replicates around the most distinctive features of the radiocarbon record to ensure our key findings were robust. Further details are provided in the manuscript and Source Data Fig. 2.
Randomization	The data for this study were acquired in no specific order or grouping, and all individual results were assessed against the same pretreatment, IRMS, and AMS quality control standards.
Blinding	The 12 samples that were were sent for replication to CEZA Mannheim were supplied without any information about the origin or expected age.

Reporting for specific materials, systems and methods

We require information from authors about some types of materials, experimental systems and methods used in many studies. Here, indicate whether each material, system or method listed is relevant to your study. If you are not sure if a list item applies to your research, read the appropriate section before selecting a response.

Materials & experimental systems

n/a	Involved in the study	
☒	☐	Antibodies
☒	☐	Eukaryotic cell lines
☐	☒	Palaeontology and archaeology
☒	☐	Animals and other organisms
☒	☐	Human research participants
☒	☐	Clinical data
☒	☐	Dual use research of concern

Methods

n/a	Involved in the study	
☒	☐	ChIP-seq
☒	☐	Flow cytometry
☒	☐	MRI-based neuroimaging

Palaeontology and Archaeology

Specimen provenance	The four samples used in this study had Canadian Heritage Parks Canada catalogue numbers 4A 59 E3-1, 4A 68 E2-2, 4A 68 J4-6 and 4A 70 B5-14. A Collections and Transfer Registration form was authorized by Parks Canada Agency official Kevin Jenkins and signed upon reception by researcher Dr Margot Kuitems on 9/11/2018 at Halifax, Nova Scotia.

Specimen deposition	The main items were returned to the official Parks Canada Storage after sampling. The excess material after analysis is held at CIO, Groningen. Requests for sample material can be made to the corresponding authors.
Dating methods	The radiocarbon dates were obtained on alpha-cellulose extracts taken from the above-mentioned samples at CIO (Groningen) and CEZA (Mannheim). The pretreatment, measurement and analytical methods employed by both laboratories are all described in full in the main text. Data quality was ensured by the use of internal and external standards (e.g. tree ring material from 1503 CE, UK; background wood from a Pleistocene deposit Kitzbühel, Austria; NIST oxalic acid II; Merck™ caffeine; IAEA C7 and C8). Results were calibrated using OxCal 4.4 against IntCal20.

☒ Tick this box to confirm that the raw and calibrated dates are available in the paper or in Supplementary Information.

Ethics oversight	Approval to undertake the study was given by the University of Groningen. Approval to take and analyse the samples, and publish the results, was obtained from Government of Canada, Parks Canada Agency.

Note that full information on the approval of the study protocol must also be provided in the manuscript.
